# Precision medicine review: rare driver mutations and their biophysical classification

**DOI:** 10.1007/s12551-018-0496-2

**Published:** 2019-01-04

**Authors:** Ruth Nussinov, Hyunbum Jang, Chung-Jung Tsai, Feixiong Cheng

**Affiliations:** 1 Cancer and Inflammation Program, Leidos Biomedical Research, Inc., Frederick National Laboratory for Cancer Research, National Cancer Institute, 1050 Boyles St., Frederick, MD 21702 USA; 2 Department of Human Molecular Genetics and Biochemistry, Sackler School of Medicine, Tel Aviv University, 69978 Tel Aviv, Israel; 3 Genomic Medicine Institute, Lerner Research Institute, Cleveland Clinic, Cleveland, OH 44106 USA; 4 Department of Molecular Medicine, Cleveland Clinic Lerner College of Medicine, Case Western Reserve University, Cleveland, OH 44195 USA; 5 Case Comprehensive Cancer Center, Case Western Reserve University School of Medicine, Cleveland, OH 44106 USA

**Keywords:** Conformational ensembles, Phenotypic drug discovery, Ras, KRas, Signaling pathways, Pharmacology, Deep sequencing, Genomics, Proteomics, Drug discovery, Machine learning

## Abstract

*How can biophysical principles help precision medicine identify rare driver mutations*? A major tenet of pragmatic approaches to precision oncology and pharmacology is that driver mutations are very frequent. However, frequency is a statistical attribute, not a mechanistic one. Rare mutations can also act through the same mechanism, and as we discuss below, “latent driver” mutations may also follow the same route, with “helper” mutations. Here, we review how biophysics provides mechanistic guidelines that extend precision medicine. We outline principles and strategies, especially focusing on mutations that drive cancer. Biophysics has contributed profoundly to deciphering biological processes. However, driven by data science, precision medicine has skirted some of its major tenets. Data science embodies genomics, tissue- and cell-specific expression levels, making it capable of defining genome- and systems-wide molecular disease signatures. It classifies cancer driver genes/mutations and affected pathways, and its associated protein structural data guide drug discovery. Biophysics complements data science. It considers structures and their heterogeneous ensembles, explains how mutational variants can signal through distinct pathways, and how allo-network drugs can be harnessed. Biophysics clarifies how one mutation—frequent or rare—can affect multiple phenotypic traits by populating conformations that favor interactions with other network modules. It also suggests how to identify such mutations and their signaling consequences. Biophysics offers principles and strategies that can help precision medicine push the boundaries to transform our insight into biological processes and the practice of personalized medicine. By contrast, “phenotypic drug discovery,” which capitalizes on physiological cellular conditions and first-in-class drug discovery, may not capture the proper molecular variant. This is because variants of the same protein can express more than one phenotype, and a phenotype can be encoded by several variants.

## Introduction: precision pharmacology faces challenges

Drug development has been facing challenges. Recent statistics quoted 44% of the discontinued phase III drugs as being due to inadequate efficacy, and 24% safety issues (Mills 2016). Several reasons were proposed as drivers of failure (Grignolo and Pretorius 2016): inadequate basic science, suboptimal dose selection, insufficient assessment of the standard of care and of disease area landscape, flawed study design, flawed data collection/analysis, and study operations problems. Recent publications argued that even though improvements in medicinal chemistry should have led to higher reliability and reproducibility, that is not the case due to irreproducibility of the results (Begley and Ellis 2012; Ioannidis 2005; Perrin 2014; Prinz et al. 2011) and higher failure rates in clinical trials (DiMasi 1995; Hay et al. 2014) as well as higher R&D costs (Booth and Zemmel 2004; Munos 2009; Scannell et al. 2012; Scannell and Bosley 2016).

The advent of precision medicine aims to reset the pharmacology button (Cheng et al. 2019). The NIH-funded thrust “to catalogue and discover major cancer-causing genome alterations in large cohorts… through large-scale genome sequencing and integrated multi-dimensional analyses” (Tomczak et al. 2015), coupled with individual scientist-inspired research and pharmaceutical companies’ efforts, has propelled the drug discovery frontiers with innovative, data- and concept-driven approaches. The vast amount of generated data along with advanced, more powerful computational strategies for its analysis and interpretation promise to transform diagnosis and cure. Precision medicine abstracts genome signatures and considers cell, tissue, and clinical observations, recently made even more powerful by high-throughput time-series analyses that permit following cancer cell evolution (Cheng et al. 2019). The recent TCGA (The Cancer Genome Atlas) collection tells what appears to be a success story (Welcome to the Pan-Cancer Atlas 2016). Its three categories, embracing patterns in cells-of-origin (Hoadley et al. 2018), oncogenic processes (Ding et al. 2018), and signaling pathways (Sanchez-Vega et al. 2018), illustrate depth and breadth. Among the key emerging questions looms one of the most important to human health: how to leverage this information in drug discovery. This challenge has led the TCGA teams to focus on patterns of cancer vulnerabilities that will help in new combination therapies. Among these are individual and co-occurring actionable alterations in prominent cancer pathways including those involved in *MYC*, *RAS*, ubiquitin, DNA damage repair, splicing, and metabolism (Ge et al. 2018; Knijnenburg et al. 2018; Peng et al. 2018; Schaub et al. 2018; Way et al. 2018). The teams are large and multidisciplinary. They include experimental and computational biologists, basic sciences, and the clinics. The methods are diverse and comprehensive and span disciplines. They include analysis of genomes, transcriptomes, and proteomes; they also encompass analysis of protein structures to more reliably identify driver mutations, commonly defined as statistically frequent mutations that drive cancer.

Nonetheless, biophysical principles have largely been skirted (Nussinov and Wolynes 2014). Biophysics has been called “the bridging science.” Biophysicists seek to explain complex biological phenomena. Modern technologies produce huge amounts of data. To arrive at conclusions and make predictions, biophysics considers the fundamental underpinnings of the constituents of cells and tissues, their dynamic environments, and the communication between the molecular entities. Even though to date biophysics has been applied increasingly to the biological sciences, this has not been the case in the emergent branch of precision medicine, despite its ability to help decipher enigmatic questions, such as how changes in the DNA of healthy cells can trigger their transformation into cancer cells and how exactly pathogens can trigger cancer and neurodegenerative diseases, such as Parkinson (Nussinov et al. 2014).

Here, we offer some principles and guidelines for extending precision medicine through biophysics. Key among these principles is that protein structures exist as conformational ensembles which can be expressed as phenotypic traits (Akhter and Shehu 2018; Alhadeff et al. 2018; Cukier 2018; Frauenfelder et al. 1991; Gunasekaran et al. 2004; Jang et al. 2016a, b; Jenkins et al. 2018; Kumar et al. 2000; Mickert and Gorris 2018; Naganathan 2018; Nguemaha et al. 2018; Nussinov 2016; Nussinov et al. 2017; Nussinov et al. 2016, 2018a; Qiao et al. 2018; Tsai and Nussinov 2018). We consider how a mutation can affect phenotypic traits by linking to different network modules and, especially, how such conformational effects may extend and empower innovative drug discovery concepts. Notably, this applies to frequent and rare mutations.

Successful identification of drug candidates with either single cell or several cell types, as compared with targeted discovery, has reinvigorated phenotypic drug discovery (Giuliani et al. 2018; Heilker et al. 2018; Vaidya et al. 2018). At first glance, the re-emergence of the phenotypic drug discovery discipline is anathema to genome-based precision drug discovery, where a panoply of mutations, or genomic aberrations, can be expressed by similar disease phenotypes at the cellular or organism level. However, phenotypic drug discovery can produce first-in-class drugs and assess a target in its physiological cell environment. As such, it can complement precision medicine. Nonetheless, it is unable to reliably target the underlying mutational origin while minimizing toxicity.

## Nomenclature/definitions

Here, we clarify some of the terminology used below:

### Orthosteric site

The functional site, e.g., active sites for enzymes, or protein-protein binding sites.

### Allosteric site

A site away from the orthosteric site but whose perturbation by binding will affect the conformation at the orthosteric site.

### Conformational distributions

The distributions of distinct conformations of the ensemble in the free energy landscape, which display their relative populations (i.e., corresponding free energy levels).

### “OR,” incremental “graded,” “AND” all-or-none logic gate operations

For simplicity, we explain these logic gates with kinase examples (Bradshaw 2010). In an “OR” logic gate switch, one event is sufficient for complete enzyme activation (e.g., Syk kinase is an “OR-gate switch” since either phosphorylation OR ITAM binding is sufficient to fully activate it). In a “graded” switch, there is an incremental increase in kinase activity with an increasing number of activating stimuli. Unlike a “graded” switch, an “all-or-none” switch has only two possible responses: either inactive or fully active (Bradshaw et al. 2003; Lisman and Zhabotinsky 2001; Ninfa and Mayo 2004). Thus, an “all-or-none” switch can be either an “OR” gate or an “AND” gate. In an “OR” gate, a single stimulus is sufficient for full activation; however, an “AND” gate requires multiple stimuli (Prehoda and Lim 2002); Tec kinase activation is an “AND” gate switch (Bradshaw 2010). The type of gate-switching mechanism is decided by the kinase features. In Tec kinases, both phosphorylation of the activation loop and the interaction with the SH2 linker are required.

## How biophysics can empower concepts and approaches in precision medicine

With the same phenomena, biophysicists and biologists ask different questions (Bialek 2011). To biophysicists, mechanisms can only be fully understood at the detailed structural level. Pathway diagrams are informative and important, but insufficient. Biophysicists venture into microscopic scales. They seek to understand why evolution has selected a certain presumably optimal solution to a problem that is essential in the life of the organism, and how it is reached and tuned—with the adage that if they are able to understand, they may be able to predict the observable behavior and exploit it in design. To biophysicists, understanding implies comprehending the molecular behavior on the conformational level.

**Fig. 1:**
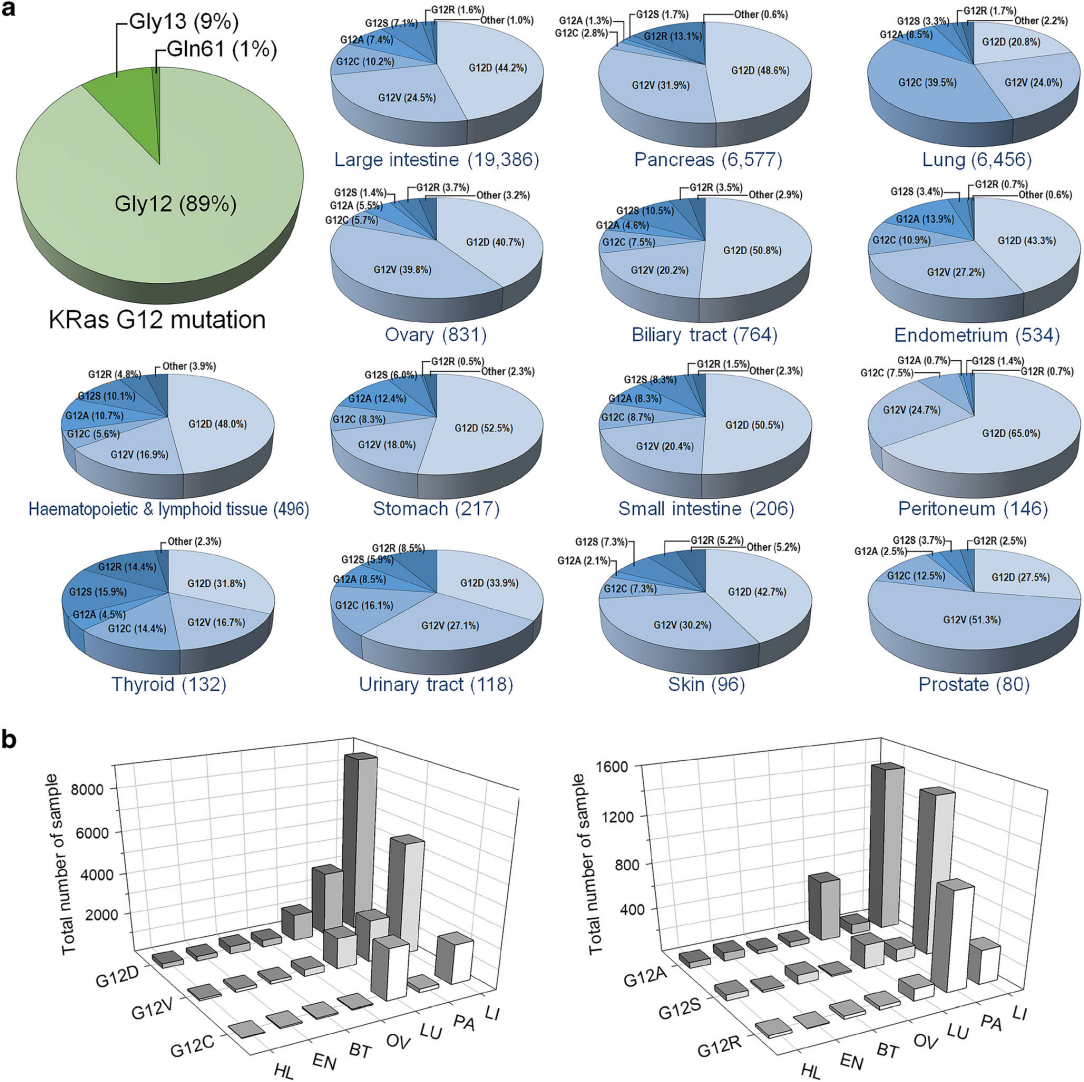
Examples of driver mutations in *KRAS*-driven cancer. **a** KRas is the most highly mutated Ras isoform in cancer. Among the oncogenic mutations at the active site, Gly12 is the most highly populated (89%). Gly13 (9%) and Gln61 (1%) (large pie at the top left corner) display lower frequencies. For Gly12, the proportions of occurrences of six different driver mutations, G12D, G12V, G12C, G12A, G12S, and G12R, are shown for 14 different cell/tissue types. The numbers in parenthesis indicate the total number of mutated samples taken from the Catalogue of Somatic Mutations in Cancer (COSMIC) database. Gly12 alterations also include rare mutations, such as G12E, G12F, G12I, etc. **b** Distributions of a mutated sample of seven tissue types, the large intestine (LI), pancreas (PA), lung (LU), ovary (OV), biliary tract (BT), endometrium (EN), and hematopoietic and lymphoid tissue (HL) for the three major KRas Gly12 driver mutations, G12D, G12V, and G12C (left panel), and three minor mutations, G12A, G12S, and G12R (right panel)

Precision, or personalized, medicine rests primarily on individual genomic sequences (Broes et al. 2018; Caskey 2018; Hampel et al. 2017; Hyman et al. 2017; Martin et al. 2015; Nakagawa and Fujita 2018; Poulos and Wong 2018; Prasad 2016; Senft et al. 2017; Tannock and Hickman 2016; Vargas and Harris 2016; Voest and Bernards 2016; Yu et al. 2017). The variability in human DNA is large, and the paramount question becomes how to identify among the many variations in the genomic sequences those which cause—or can cause—susceptibility to the disease. Standard approaches involve a priori identification of driver mutations, i.e., those whose association with the disease is statistically significant (Fig. 1 provides an example for *KRAS* mutations). If the statistical significance of a mutation is high, its correlation with the disease is indicated. These driver mutations are then compiled and stored in databases. Subsequent observations of these mutations in an individual can testify to the origin of the illness (Raphael et al. 2014; Tsang et al. 2017).

However, statistics cannot explain *how* these mutations elicit the observed phenotype (Barone et al. 2017; Bilal et al. 2013; Blucher et al. 2017; Hogeweg 2011; Mathew et al. 2007; Payne 2012; Winter et al. 2012; Yakhini and Jurisica 2011). It also falls short when disease-promoting mutations are rare. Structural knowledge can help in both identifying mutations with low statistical significance and revealing the mechanism behind those statistically significant (Raphael 2012). The first step would involve mapping the mutations onto their corresponding macromolecular structures to identify their location and structural environment, and uncovering the interactions the protein is involved in, and which the mutations may affect. The mutations would be analyzed with respect to *how* they may affect these interactions and thereby rewire the cellular network. If located in highly flexible, or disordered, regions, the chances of their driver character are higher. Residue type may also help in providing clues: chances are higher if the mutated residue is aromatic or charged. Such regions are more likely to play a role in conformational changes with the altered interactions shifting the relative stabilities, typically of the inactive in favor of the active conformations. Mutations involving charged or aromatic residues are also likely to lead to a similar outcome. Eventually, the networks of protein-protein and protein-DNA (or RNA) interactions determine cell behavior. Importantly, for membrane-interacting proteins, the mutations should be considered within this framework as well. Hydrophobic or charged residues may be particularly involved in altered membrane-interaction tendencies. Interactions with signaling lipids such as eicosanoids, phosphoinositides, sphingolipids, and fatty acids control critical cellular processes, including cell proliferation, apoptosis, metabolism, and migration (Wymann and Schneiter 2008). Mechanistic consequences may also involve mutations of residues which can be post-translationally modified (PTMs). PTM effects can be either via an allosteric mechanism (Nussinov et al. 2012), or by abolishing or altering direct molecular interactions, thereby shifting an inactive protein state to an active one, or vice versa.

Altogether, mechanistic underpinnings that decide the mutational effects—whether statistically frequent or rare, at the tail of the distribution—can involve orthosteric or allosteric effects (Cheng and Nussinov 2018; Cheng et al. 2016; Collier and Ortiz 2013; Feher et al. 2014; Fetics et al. 2015; Liu and Nussinov 2008; Lu et al. 2015, 2016a, b; Marcus and Mattos 2015; Morra et al. 2009; Munro et al. 2018; Nikolaev et al. 2018; Park et al. 2018; Risques and Kennedy 2018; Shen et al. 2017; Stout and Campbell 2018; Tehver et al. 2009; Tsai and Nussinov 2014, 2017; Tuncbag et al. 2017; Waters and Der 2018; Xu et al. 2017; Zhan et al. 2016). Orthosteric mutations are at binding or active sites and can directly abolish (most frequent) or strengthen (much less frequent) interactions. The interactions can be between proteins, between the protein and nucleic acids, and between the protein and the membrane. Allosteric effects can rewire the cell network via short- or longer-range effects. Notably, all involve a shift in the conformational ensemble, including orthosteric mutations, since they too disturb the structure and elicit changes in the atomic interactions. Network rewiring reflects the changes in the conformational distributions whose outcome may favor different partners at shared binding sites (Boehr et al. 2009; del Sol et al. 2009). The shift of the ensemble can also explain the propagation of signaling, i.e., “allo-networks” (Nussinov et al. 2011; Szilagyi et al. 2013). As we have postulated earlier, “allosteric signal propagation does not stop at the ‘end’ of a protein; but may be dynamically transmitted across the cell.” The signaling can be transmitted within a macromolecule, such as protein, DNA, or RNA, or across interacting molecules, enhancing or inhibiting specific interactions as the “allosteric wave” propagates.

The challenge in the identification of mutations and the complexity involved in their mechanisms, which is expected to involve combinations of mutations within the same or different proteins whose conjoint effects may alter the network, can be strikingly observed from recent statistics (Alexov 2014). The 1000 Genomes pilot project (Clarke et al. 2012) indicated that most individuals carry 250 to 300 loss-of-function mutations in annotated genes with 50–100 mutations identified earlier as likely to be involved in inherited disorders (Genomes Project et al. 2010). Critically, the mutational load and the cell/tissue environment affect the severity of a disease. These may reflect the level of expression, protein and RNA concentrations, metabolites, presence/absence of microbiota that can mitigate or aggravate disease phenotypes, and more. The emerging complexity and ramifications pose an enormous challenge. In the absence of good biophysical models, exactly how to incorporate thousands of genetic variations of proteins is, however, unclear. While feasible, combining individual gene variability may lead to poor predictors of disease.

## “Latent driver” mutations, statistically rare mutations, and the cellular network

**Fig. 2:**
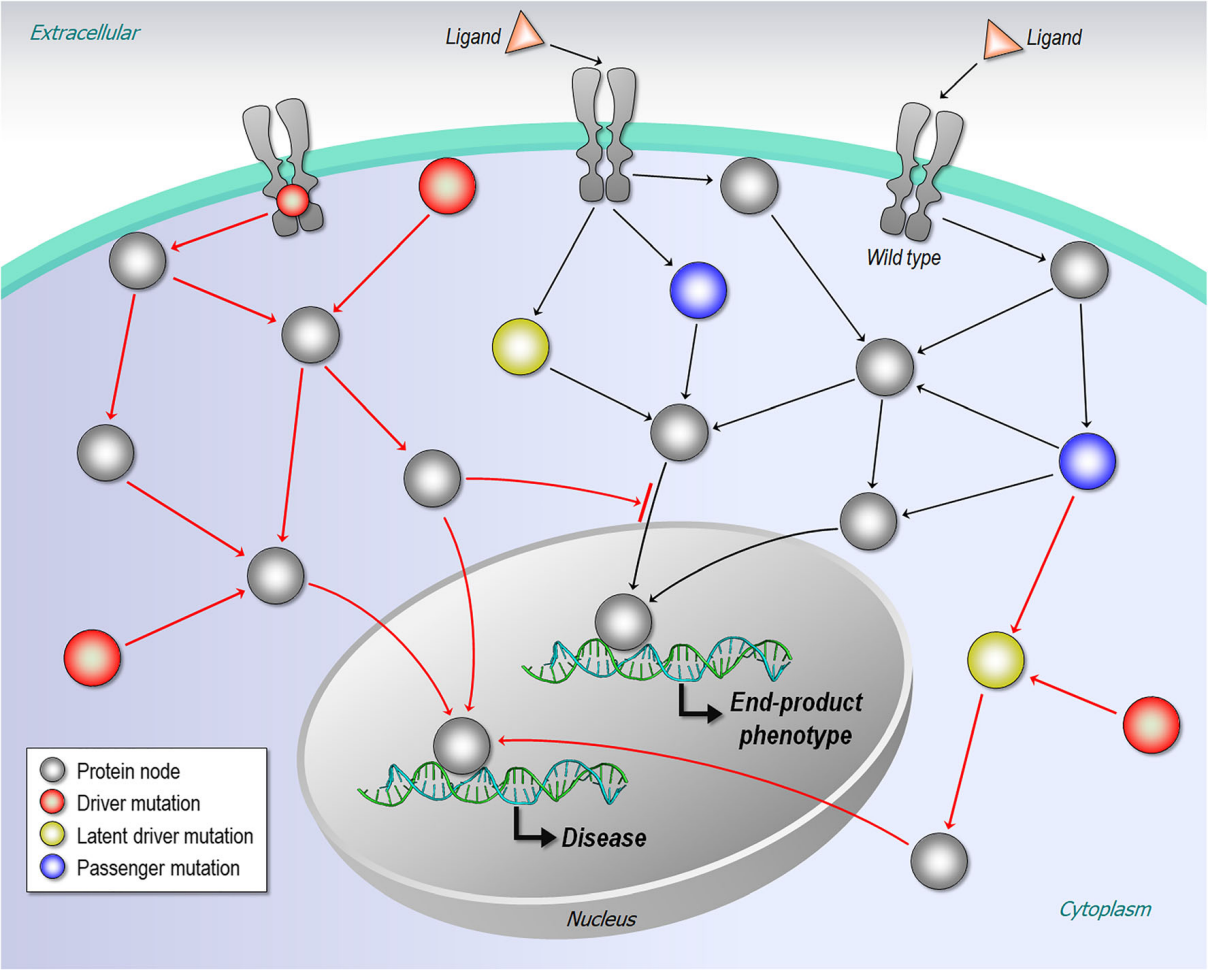
Mutations in the cellular network. In diseases, driver mutations affect the cellular network to alter phenotypic traits. A latent driver mutation, like a passenger mutation, does not confer a cancer cell phenotype. However, together with a newly evolved mutation, it can express a cancer cell phenotype

Although many mutations are inherited, most appear during normal cell growth (Nussinov and Tsai 2015; Vogelstein et al. 2013; Welch et al. 2012). Most of these are considered “passengers,” as are most of those emerging during cancer evolution. By contrast, “driver mutations,” whether inherited or gained, acquire certain hallmarks of cancer, thereby conferring cancer cell advantage, including drug resistance (Fig. 2) (Egeblad et al. 2010; Hanahan and Weinberg 2000, 2011; Merlo et al. 2006; Nussinov and Tsai 2013; Thiagalingam et al. 1996). While mutation rates vary (Bignell et al. 2010; Stratton et al. 2009; Youn and Simon 2011), a puzzling question has been why a primary cancer can develop over many years whereas drug resistance develops rapidly (Nussinov and Tsai 2015). Even though resistance mechanisms are diverse (e.g., Knievel et al. 2014; Nakamura et al. 2013; Poulikakos et al. 2010; Roskoski Jr. 2014), “latent driver” mutations may be among them. Like a passenger mutation, on its own a latent driver mutation does not confer a cancer hallmark; however, together with a newly evolved mutation, it can express a cancer cell phenotype and cause drug resistance. A latent driver mutation can rewire the cellular network via “AND” all-or-none or incremental “graded” logic gate operations (Nussinov and Tsai 2015). Since, to date, “actionable mutations” have only been those identified by their statistically significant frequencies (Cheng et al. 2019), latent driver mutations have been overlooked.

The “latent driver” concept builds on a conformational view of macromolecules. According to this view, a macromolecule has a certain distribution of its ensemble, which reflects its environment. An environment can include concentration; sequence; post-translation modifications; interactors, such as proteins, nucleic acids, lipids, and small molecules; pH; temperature; ions; the crowded cellular environment; etc. A mutation may stabilize or destabilize the native state. The extent of the stabilization/destabilization with respect to the wild type may be minor, in which case the phenotypic change may not be observed; however, integrated with an emerging mutation whose contribution may also be minor, it may promote cell transformation or drug resistance. Together, the mutations can incrementally shift the protein ensembles toward populating a constitutively active (or inactive for a repressor protein) state (Tsai and Nussinov 2014), eliciting a cancer hallmark. Identifying such mutational partners is highly challenging.

Rare mutations can drive cancer development even if they are at the tail of the distribution. Even though not identified as drivers, the mechanisms of these mutations resemble those of mutations which are frequent and thus identified as drivers. Like statistically significant mutations, rare drivers can occur at the binding site, thus directly abolishing binding or allosterically shifting the protein ensemble from an *inactive* to an *active* state (or vice versa). Here, too, the challenge is their identification, pulling them out of the background passenger mutational clutter.

A protein (node) in a signaling pathway is typically modulated by upstream activation (or inhibition), with the effects (transmission of the signals or blocking them) propagating via interactors to pathways downstream, thereby affecting the network (Fig. 2). At the basic, conformational level, the modulation switches the population of the ensemble. In the absence of an upstream signal, passenger mutations cause no change in the relative (active versus inactive) populations, thus no downstream signaling shift. Even if the effects of several passenger mutations are integrated, there is still no change toward the distinct active conformation. This contrasts with driver and latent driver mutations. Both lead to alterations in the populations of the active vs inactive state—however to different extents. Latent drivers would shift the distributions in favor of the active (or inactive) state; however, the extent is insufficient for an observable phenotypic change. On the other hand, by definition, a driver mutation would result in a clear observable change. A rare driver is a driver, except that its statistics is lower. Heterogeneous protein ensembles can explain how mutations can elicit the observed phenotype; thus, the definitions of driver, latent driver, passenger, and rare driver hinge on their change of relative conformational populations between the active and inactive states with respect to that of the wild type and their statistics.

Taken together, we can view protein molecules in terms of their conformational ensembles; this contrasts with a network view which perceives proteins as nodes in networks. Considering research disciplines, chemists aim to obtain drugs that target actionable mutants; mathematicians and physicists consider the consequences of the inhibition on the cellular networks which they view as nodes and edges (the interactions connecting them). However, allosteric propagation across the protein, and via its interactors to the pathways, merges the two views and thereby can extend the database of drug targets (Csermely et al. 2013; Szilagyi et al. 2013). The propagation of allosteric signals can dynamically transcend their interfaces. However, we would still not expect the signal to travel a long way, through multiple pathway-associated proteins. For this to take place, there should be additional perturbations downstream. Binding to a protein may elicit distance-limited, specific, and network-neighboring changes (Antal et al. 2009; Ma and Nussinov 2009; Nussinov et al. 2011; Tsai et al. 2009).

Protein ensembles link physicochemical principles with the cellular network and protein behavior in vitro and in vivo. As we discuss below, they are also critical components in pharmacogenomics and pharmacogenetics.

## Driver mutations, interactions, and cell signaling

**Fig. 3:**
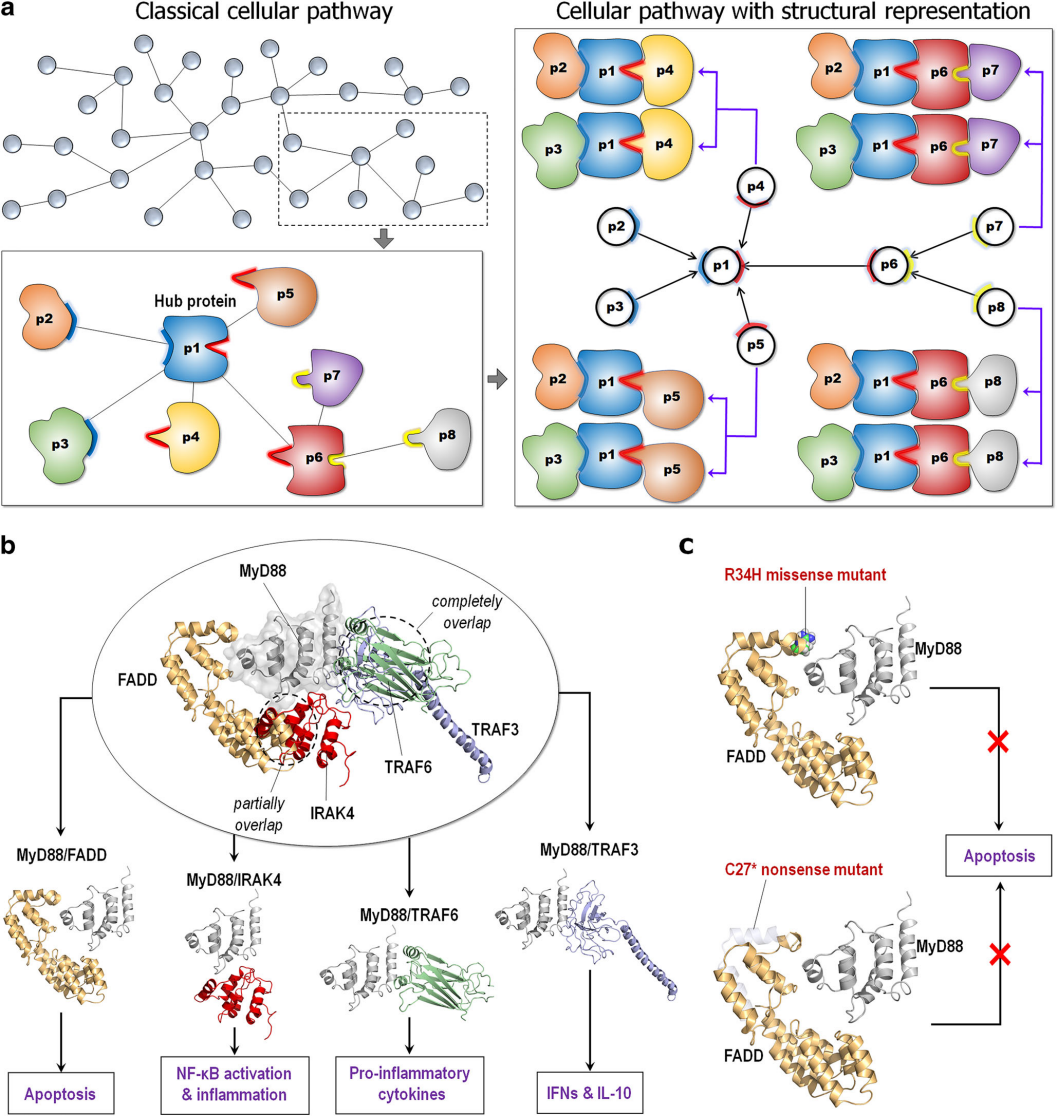
The protein interaction network and its mutational effects. **a** A classical representation of a cellular pathway (top left). In the cellular network, proteins (nodes) connect with each other through protein-protein interactions (edges). Node traits can be highlighted by structural representation in the cellular pathway (lower left panel). Hub proteins provide shared binding sites for many binding partners. Edge traits can be characterized by specific combinations of the protein-protein interactions (right panel). The cartoons were inspired by a previous publication (Tuncbag et al. 2009). **b** An example of the hub protein MyD88 (PDB code: 3MOP) and its binding partners. FADD (PDB code: 2GF5) and IRAK4 (PDB code: 3MOP) partially overlap at the same binding site of MyD88, and TRAF6 (PDB code: 1LB5) and TRAF3 (PDB code: 1FLL) share the same binding site of MyD88 and completely overlap at the interface. These proteins compete to bind to MyD88, causing distinct downstream signaling pathways. **c** Mutations can alter edge traits. Examples are shown for R34H missense and C27* nonsense mutants of FADD. These mutations abolish the interaction of FADD with MyD88, rewiring cellular network. Protein complex constructions were inspired by a previous publication (Guven-Maiorov et al. 2015)

Driver mutations can destabilize or stabilize proteins, shifting their states between active (or ON) or inactive (OFF) states. They can also rewire cell signaling. Identifying those mutations that shift states can help identify rare driver mutations. Key proteins, especially hubs, are epitomized by shared binding sites (Fig. 3a) (Gursoy et al. 2008; Tuncbag et al. 2009). Mutations that abolish an interaction of a partner protein binding at a shared site can favor binding of another protein at that site, thereby rewiring the network, which may now favor, e.g., pro-inflammatory signaling rather than apoptosis (Fig. 3b) (Guven-Maiorov et al. 2015). One example is the case of C27* mutation in the Fas-associated protein with death domain (FADD) protein in lung squamous carcinoma (0.21%, TCGA), which our data (unpublished) suggest can abolish its interaction with myeloid differentiation factor 88 (MyD88), permitting interleukin-1 receptor-associated kinase 4 (IRAK4, the NF-κB pathway) to occupy that MyD88 site (Fig. 3c). Similarly, TNF receptor-associated factor 6 (TRAF6) and TRAF3 occupy overlapping binding sites on MyD88; thus, MyD88 induces either pro- or anti-inflammatory cytokines. A R505* TRAF3 mutation in head and neck tumors (4.7% frequency) abolishes its binding to MyD88, which promotes TRAF6 binding, possibly explaining how it contributes to cancer.

Mutations can shift not only the preferred binary interactions but also interactions in multimolecular assemblies (or “signalosomes”), and in this way alter cell signaling. This can alter the intramolecular crosstalk (Rowland et al. 2017) and influence drug activity. Structural data, coupled with efficient structural comparison algorithms, appropriate datasets, and filters can assist in getting an insight into such shared sites, and in obtaining concrete predictions of interactions consistent with experiment. Mutations that favor such a switch, even if rare, are likely to be drivers. Driver mutations can alter the regulation of the signalosome assembly/disassembly. Notably, when inhibited, the impacts on different tissues will vary, indicating that cellular context is crucial for targeting signaling networks.

## Conformational heterogeneity can help bridge pleiotropy and pharmacogenetics

As an example for how biophysics can help link pharmacogenomics with precision medicine, consider the emerging principle that variation in any one pharmacogene may impact the clinical outcome for more than one drug (Oberg et al. 2016), since one gene often impacts more than one clinical outcome. This can be straightforwardly understood in terms of a shift in the ensemble, leading to an altered favored interacting partner. It can also reflect an altered pattern of post-translational modifications in distinct tissues. Notably, according to the standard definition, unlike pharmacogenomics, pharmacogenetics considers how variation in a single gene influences the response to a single drug.

**Fig. 4:**
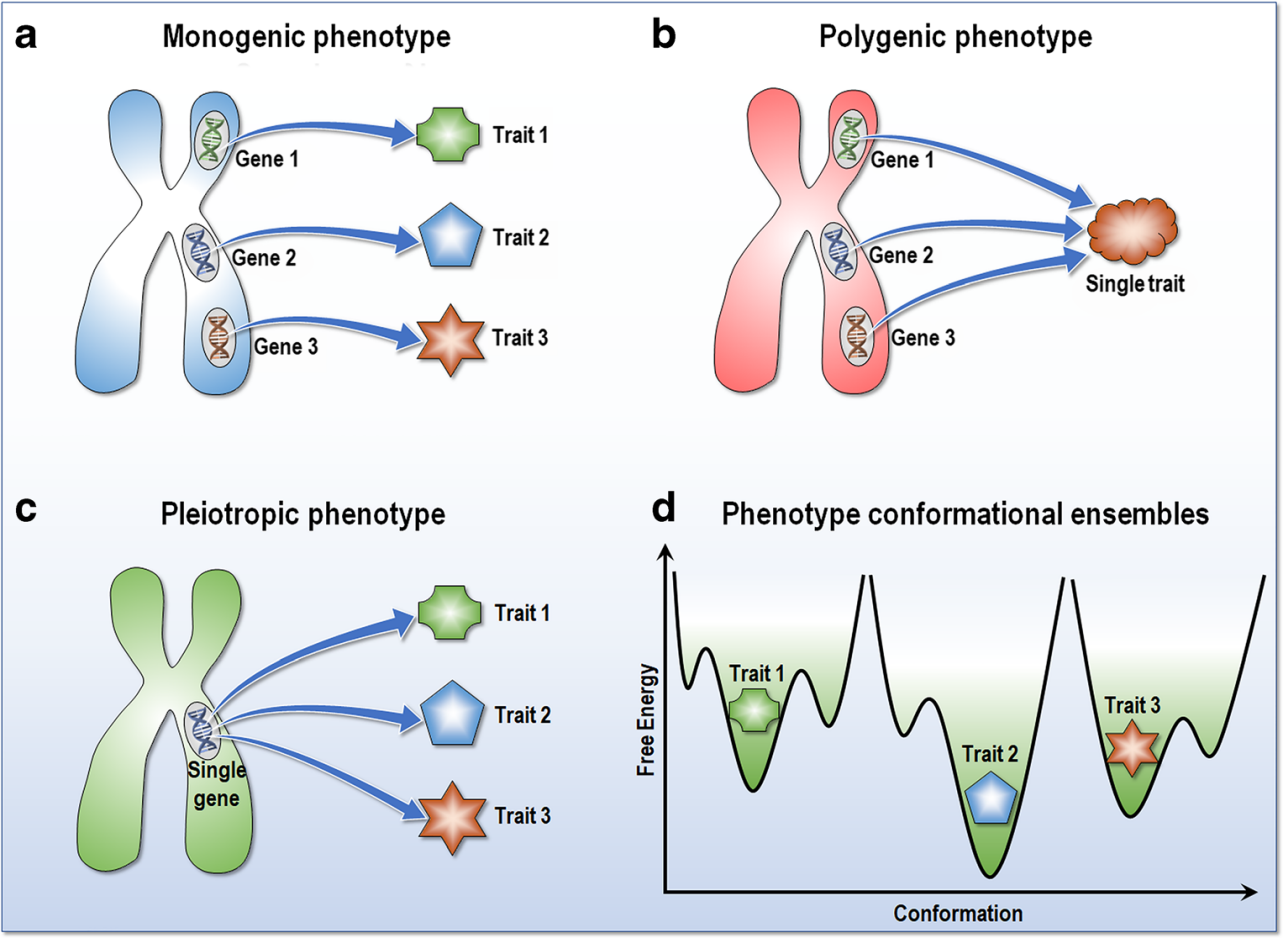
Genotype and phenotype. **a** Monogenic phenotype occurs when a single gene expresses each trait, **b** while polygenic phenotype arises when multiple genes influence a single trait. **c** Pleiotropy occurs when seemingly unrelated phenotypic traits are expressed by a single gene. **d** In a new genotype-phenotype paradigm, a pleiotropic gene can encode a distinct conformational ensemble in all states with certain populations of the specific phenotype traits that link to genotype. See text for further details

Thus, even though classically not described in this light, pleiotropy can reflect the presence of multiple states (Fig. 4). While definitions vary, a pleiotropic gene (or genetic variant) is often conceived as affecting multiple traits (Hodgkin 1998; Houle et al. 2010; Solovieff et al. 2013; Stearns 2010; Tyler et al. 2016; Wagner and Zhang 2011; Wang et al. 2010), as for example in the case of the small heat shock family α-crystallin B chain R120G mutation in mice which causes cataracts and cardiomyopathy (Andley et al. 2011). Pleiotropic genes that affect related traits cluster into relatively independent modules, with a mutation more likely to have smaller effects on unrelated traits in a different module than on related traits (Pendergrass and Ritchie 2015; Wagner and Zhang 2011). Comprehensive phenome-wide association study (PheWAS) of the National Health and Nutrition Examination Surveys (NHANES) observed potential pleiotropic genes with 13 SNPs associated with more than one phenotype (Hall et al. 2014), and a broad analysis of disease-causing proteins in UniProt revealed that 12% are pleiotropic, i.e., variants in the same protein cause more than one disease (Ittisoponpisan et al. 2017). Pleiotropy was also suggested as a reason why a drug that targets only one phenotype may fail, and thus, developing drugs that target only one phenotypic effect of a pleiotropic gene could be futile (Wagner and Zhang 2011). Classically, pleiotropy was captured in cases of clear, observable linkage between disease and a distinct mutation. Biophysics can extend such observations, offer their underlying mechanisms, and bridge the disciplines. A major tenet of biophysics is that a single mutation—frequent or rare—or another allosteric event can induce different phenotypic traits by populating conformations within the same or different network modules, and favor certain signaling pathways.

## Phenotypic drug discovery

Phenotypic drug discovery has been dubbed “classical pharmacology,” which is the historical basis of drug discovery—in contrast to “reverse pharmacology,” which is considered as target-based drug discovery (Lage et al. 2018). The classical concept of “phenotypic drug discovery” is thus not new. However, questions about the sustainability of the current target-based drug discovery process have triggered its renaissance which capitalizes on physiological cellular conditions and first-in-class drug discovery, and a recent perspective attempted to merge the phenotypic drug discovery concept with a target-based view (Heilker et al. 2018). The root of the phenotypic drug discovery concept is an anathema to the structure-based biophysics view, as well as to precision medicine whose basis is specific genomic sequences and mutations. Phenotypic drug discovery is now considered in databases, with assays that it employs grouped by animal disease model or phenotypic endpoint, accounting for assay data on protein targets or cell- or tissue-based systems (Hunter et al. 2018). Accounting for phenotypic consideration was also proposed to help computationally guided drug repurposing (Giuliani et al. 2018). Phenotypic screens were also proposed to be useful in chemogenomics (Jacoby and Brown 2018), as well as bridging the gap between phenotypic and biochemical assays (Denny 2018). Exploiting image-based profiling assays for assessing single-cell phenotypes has also been explored, including by machine learning (Scheeder et al. 2018). Powerful cell-based assay technologies that permit tighter linkage between in vitro and physio-pathological conditions and environments, including induced pluripotent stem cells, three-dimensional models, co-culture, and organ-on-a-chip systems, as well as advances in gene-editing technologies (Dorval et al. 2018), were put forward as the reasons for this renaissance and its promising future. Methods have also been developed to capture it (Joslin et al. 2018). These have triggered excitement for phenotypic drug discovery approaches, which can complement large-scale drug discovery efforts. It was further suggested that some diseases would benefit from a focus on a relevant condition or phenotype, rather than a specific target, via coupling network pharmacology with phenotypic screening (Sidders et al. 2018). A recent review provides an excellent overview of these and other small-molecule discovery technologies with examples (Markossian et al. 2018), and comparative analyses have been carried out as well (Riddy et al. 2018). A search of the literature yields a plethora of phenotypic drug discovery approaches, developments, and applications (e.g., Ayotte et al. 2018; Chatelain and Ioset 2018; Lagunin et al. 2018; Lane et al. 2018; Orellana et al. 2018; Ortiz et al. 2017), and finally, a cautionary note was also put forward (Copeland and Boriack-Sjodin 2018).

From the biophysical standpoint, protein (i.e., target) variability, even that induced by a single mutation or a change in the environment, may elicit altered phenotypes. The converse also holds: an observed phenotype, such as a disease, may reflect not only different proteins for example in the same signaling pathway but also variants of the same proteins. A phenotype may have diverse origins, and thus, even though the phenotypic approach may be helpful under certain circumstances in speeding up discovery, it may or may not capture the proper variant and accurately predict its source. The heated controversy between the supporters of phenotypic and target-focused screening, which is at the core of precision medicine, as to which provides a more reliable path to successful drug development is expected. Even though attempts to reconcile the two have been advocated, the biophysics axiom argues that no matter how efficient phenotypic methods are, a phenotype may not equate to a single molecular shape. Phenotype-based discovery does not identify the pathway or the protein target, and since no structure is involved, it also does not permit ab initio design, or design optimization.

## How biophysics applications can identify candidate cancer driver mutations

Examples of how biophysics applications can identify cancer driver mutations include molecular dynamics simulations of the native state and the mutant state: if the mutation promotes *destabilization* of the *inactive state*, it is a candidate for a driver mutation. Such destabilization can elicit a release of an autoinhibited state, as for example in PI3K (Nussinov et al. 2018b; unpublished data), Raf, and EGFR kinases. Simulations can also reveal mutations that can deactivate an essential enzymatic action which makes the enzyme switch back into the inactive state, as in the case of Ras oncogenic mutations, e.g., Q61L and G12 mutations (Lu et al. 2016a, b; Pantsar et al. 2018). Trapping different states experimentally, as well as simulations in the case of EGFR (Ruan and Kannan 2018; Verma et al. 2018), Raf (Rukhlenko et al. 2018), and other proteins, and integrating these with activity either directly or indirectly by observing their resulting actions downstream (e.g., on phosphorylation or transcription/translation) can provide first-hand confirmation, with the detailed structural data showing exactly how the mutation works. In another recent modeling strategy, exploration of structure, energetics, and dynamics of p53, PTEN, and SMAD4 tumor suppressor proteins has also revealed that driver mutations in these proteins inactivate structurally stable residues that play a fundamental role in global propagation of dynamic fluctuations and mediating allosteric interaction network (Verkhivker 2019). p53 mutations were also shown to alter the interaction of the protein with the genome, promoting carcinogenesis, with a biophysical model for a gain-of-function mechanism that consolidates many literature observations (Stiewe and Haran 2018). Crystallography provides the key building block in such studies (Gomes et al. 2018), as also shown by TrkA (tropomyosin receptor kinase A, a nerve growth factor receptor) and broadly in activating mutations in tyrosine kinase domains in cancer (Artim et al. 2012).

## Biophysics and precision medicine: principles and strategies

A fundamental biophysical principle is that molecules are not rigid rocks and should not be considered as single entities (Nussinov and Wolynes 2014). Statistics based on such a description may be inaccurate, especially when related to disease and treatment decisions. Molecules exist as dynamic ensembles, which integrate into and enhance the dynamic nature of interactome (Alexov 2014; Bohnenberger et al. 2011; Foerster et al. 2013). Molecules fluctuate, and these fluctuations are required for cell life (Wei et al. 2016). Fluctuation involves sampling different states, some of which have distinct functions. Fluctuations are critical for enzymes to work, for a receptor to switch between states, and for the chromatin to express the right protein at the right time (Rychkov et al. 2017). Overlooking these fluctuations does not necessarily affect the identification of targetable states, although it is required in the optimization of the drug design process. However, overlooking protein conformations and their dynamics may result in omissions in identification of protein mutations, and their consequences on the dynamic cell states, as well as accurate identification of the origin of an observed phenotypic trait. Upon mutational events, altered environment, covalent or non-covalent linkages or associations, and conformational heterogeneity may lead to different favored partners at shared binding sites and explain propagation of signaling. Biophysics can explain how variants can signal through distinct pathways, and how allo-network drugs can be harnessed. It clarifies how one mutation—frequent or rare—can affect multiple phenotypic traits by populating conformations that favor other network modules, and that such conformational effects may extend and empower innovative drug discovery concepts.

How to identify rare driver and latent driver mutations and how to construct the cellular allo-network? Mapping the mutations on the protein structure will indicate whether they adjoin functional or allosteric sites, and whether they exist in clusters, in which case they have a higher likelihood of being rare or latent drivers. Mutations that elicit a switch in partners at a shared binding site are also likely driver mutations, even if such mutations are rare, as those in highly flexible regions. Mutations that prevent execution of essential regulatory actions, such as abolishing ubiquitination of the Ras hypervariable region, thereby leading to extended membrane anchorage and mitogen-activated protein kinase (MAPK) signaling (Bigenzahn et al. 2018; Steklov et al. 2018), are also highly likely candidates. Molecular dynamics simulations might also observe changes in the distributions of the ensemble of the native vs the mutational state.

Paraphrasing the Biophysical Society (Biophysical Society 2018), one may ask what do the laws of physics, like those that define forces or energy, have to do with biology? As the Society befittingly explains, “these laws and concepts are essential to unraveling complex biological questions like how plants extract energy from sunlight and how changes in a protein’s shape affect its function… Biophysics is critical to integrating systems biology, genomics, and proteomics data into information that can guide diagnostics and medical treatment.” This is the essence of precision medicine.

Thus, while precision medicine can be viewed as driven by systems biology, and the phenotypes of diseases are complex, biophysics can help in addressing the challenge (Ali and Aittokallio 2018; Filipp 2017; Schurdak et al. 2018). Still the question looms: driver mutations are not always frequent; how to identify them? Can biophysics suggest strategies that would broaden the pharmacological targets to include not only rare driver mutations (Ciriello et al. 2013) but also latent drivers, which by definition are rare as well?

## References

[CR1] Akhter N, Shehu A (2018). From extraction of local structures of protein energy landscapes to improved decoy selection in template-free protein structure prediction. Molecules.

[CR2] Alexov E (2014). Advances in human biology: combining genetics and molecular biophysics to pave the way for personalized diagnostics and medicine. Adv Biol.

[CR3] Alhadeff R, Vorobyov I, Yoon HW, Warshel A (2018). Exploring the free-energy landscape of GPCR activation. Proc Natl Acad Sci U S A.

[CR4] Ali M, Aittokallio T (2018) Machine learning and feature selection for drug response prediction in precision oncology applications. Biophys Rev. 10.1007/s12551-018-0446-z 10.1007/s12551-018-0446-z PMC6381361 30097794

[CR5] Andley UP, Hamilton PD, Ravi N, Weihl CC (2011). A knock-in mouse model for the R120G mutation of alphaB-crystallin recapitulates human hereditary myopathy and cataracts. PLoS One.

[CR6] Antal MA, Bode C, Csermely P (2009). Perturbation waves in proteins and protein networks: applications of percolation and game theories in signaling and drug design. Curr Protein Pept Sci.

[CR7] Artim SC, Mendrola JM, Lemmon MA (2012). Assessing the range of kinase autoinhibition mechanisms in the insulin receptor family. Biochem J.

[CR8] Ayotte Y, Bilodeau F, Descoteaux A, LaPlante SR (2018). Fragment-based phenotypic lead discovery: cell-based assay to target leishmaniasis. ChemMedChem.

[CR9] Barone L, Williams J, Micklos D (2017). Unmet needs for analyzing biological big data: a survey of 704 NSF principal investigators. PLoS Comput Biol.

[CR10] Biophysical Society (2018) Becoming a biophysicist. https://www.biophysics.org/becoming-a-biophysicist. Accessed 1 Jan 2018

[CR11] Begley CG, Ellis LM (2012). Drug development: raise standards for preclinical cancer research. Nature.

[CR12] Bialek W (2011) Biophysics: searching for principles. http://www.princeton.edu/~wbialek/PHY562.html. Accessed 18 Sept 2011

[CR13] Bigenzahn JW et al (2018) LZTR1 is a regulator of RAS ubiquitination and signaling. Science. 10.1126/science.aap8210 10.1126/science.aap8210 PMC6794158 30442766

[CR14] Bignell GR (2010). Signatures of mutation and selection in the cancer genome. Nature.

[CR15] Bilal E (2013). Improving breast cancer survival analysis through competition-based multidimensional modeling. PLoS Comput Biol.

[CR16] Blucher AS, Choonoo G, Kulesz-Martin M, Wu G, McWeeney SK (2017). Evidence-based precision oncology with the cancer targetome. Trends Pharmacol Sci.

[CR17] Boehr DD, Nussinov R, Wright PE (2009). The role of dynamic conformational ensembles in biomolecular recognition. Nat Chem Biol.

[CR18] Bohnenberger H, Oellerich T, Engelke M, Hsiao HH, Urlaub H, Wienands J (2011). Complex phosphorylation dynamics control the composition of the Syk interactome in B cells. Eur J Immunol.

[CR19] Booth B, Zemmel R (2004). Prospects for productivity. Nat Rev Drug Discov.

[CR20] Bradshaw JM (2010). The Src, Syk, and Tec family kinases: distinct types of molecular switches. Cell Signal.

[CR21] Bradshaw JM, Kubota Y, Meyer T, Schulman H (2003). An ultrasensitive Ca2+/calmodulin-dependent protein kinase II-protein phosphatase 1 switch facilitates specificity in postsynaptic calcium signaling. Proc Natl Acad Sci U S A.

[CR22] Broes S, Lacombe D, Verlinden M, Huys I (2018). Toward a tiered model to share clinical trial data and samples in precision oncology. Front Med (Lausanne).

[CR23] Caskey T (2018). Precision medicine: functional advancements. Annu Rev Med.

[CR24] Chatelain E, Ioset JR (2018). Phenotypic screening approaches for Chagas disease drug discovery. Expert Opin Drug Discovery.

[CR25] Cheng F, Nussinov R (2018). KRAS activating signaling triggers arteriovenous malformations. Trends Biochem Sci.

[CR26] Cheng F, Zhao J, Zhao Z (2016). Advances in computational approaches for prioritizing driver mutations and significantly mutated genes in cancer genomes. Brief Bioinform.

[CR27] Cheng F, Liang H, Butte AJ, Eng C, Nussinov R (2019). Personal mutanomes meet modern oncology drug discovery and precision health. Pharmacol Rev.

[CR28] Ciriello G, Miller ML, Aksoy BA, Senbabaoglu Y, Schultz N, Sander C (2013). Emerging landscape of oncogenic signatures across human cancers. Nat Genet.

[CR29] Clarke L (2012). The 1000 Genomes Project: data management and community access. Nat Methods.

[CR30] Collier G, Ortiz V (2013). Emerging computational approaches for the study of protein allostery. Arch Biochem Biophys.

[CR31] Copeland RA, Boriack-Sjodin PA (2018). The elements of translational chemical biology. Cell Chem Biol.

[CR32] Csermely P, Nussinov R, Szilagyi A (2013). From allosteric drugs to allo-network drugs: state of the art and trends of design synthesis and computational methods. Curr Top Med Chem.

[CR33] Cukier RI (2018). Generating intrinsically disordered protein conformational ensembles from a database of Ramachandran space pair residue probabilities using a Markov chain. J Phys Chem B.

[CR34] del Sol A, Tsai CJ, Ma B, Nussinov R (2009). The origin of allosteric functional modulation: multiple pre-existing pathways. Structure.

[CR35] Denny PW (2018) Yeast: bridging the gap between phenotypic and biochemical assays for high-throughput screening. Expert Opin Drug Discovery. 10.1080/17460441.2018.1534826 10.1080/17460441.2018.1534826 30326751

[CR36] DiMasi JA (1995). Success rates for new drugs entering clinical testing in the United States. Clin Pharmacol Ther.

[CR37] Ding L (2018). Perspective on oncogenic processes at the end of the beginning of cancer genomics. Cell.

[CR38] Dorval T, Chanrion B, Cattin ME, Stephan JP (2018). Filling the drug discovery gap: is high-content screening the missing link?. Curr Opin Pharmacol.

[CR39] Egeblad M, Nakasone ES, Werb Z (2010). Tumors as organs: complex tissues that interface with the entire organism. Dev Cell.

[CR40] Feher VA, Durrant JD, Van Wart AT, Amaro RE (2014). Computational approaches to mapping allosteric pathways. Curr Opin Struct Biol.

[CR41] Fetics SK, Guterres H, Kearney BM, Buhrman G, Ma B, Nussinov R, Mattos C (2015). Allosteric effects of the oncogenic RasQ61L mutant on Raf-RBD. Structure.

[CR42] Filipp FV (2017). Precision medicine driven by cancer systems biology. Cancer Metastasis Rev.

[CR43] Foerster S (2013). Characterization of the EGFR interactome reveals associated protein complex networks and intracellular receptor dynamics. Proteomics.

[CR44] Frauenfelder H, Sligar SG, Wolynes PG (1991). The energy landscapes and motions of proteins. Science.

[CR45] Ge Z (2018). Integrated genomic analysis of the ubiquitin pathway across cancer types. Cell Rep.

[CR46] Genomes Project C (2010). A map of human genome variation from population-scale sequencing. Nature.

[CR47] Giuliani S (2018). Computationally-guided drug repurposing enables the discovery of kinase targets and inhibitors as new schistosomicidal agents. PLoS Comput Biol.

[CR48] Gomes AS et al (2018) The crystal structure of the R280K mutant of human p53 explains the loss of DNA binding. Int J Mol Sci 19. 10.3390/ijms19041184 10.3390/ijms19041184 PMC5979565 29652801

[CR49] Grignolo A, Pretorius S (2016) Phase III trial failures: costly, but preventable, vol 25. Iselin, New Jersey, p 08830

[CR50] Gunasekaran K, Ma B, Nussinov R (2004). Is allostery an intrinsic property of all dynamic proteins?. Proteins.

[CR51] Gursoy A, Keskin O, Nussinov R (2008). Topological properties of protein interaction networks from a structural perspective. Biochem Soc Trans.

[CR52] Guven-Maiorov E, Keskin O, Gursoy A, VanWaes C, Chen Z, Tsai CJ, Nussinov R (2015). The architecture of the TIR domain signalosome in the toll-like receptor-4 signaling pathway. Sci Rep.

[CR53] Hall MA (2014). Detection of pleiotropy through a Phenome-wide association study (PheWAS) of epidemiologic data as part of the Environmental Architecture for Genes Linked to Environment (EAGLE) study. PLoS Genet.

[CR54] Hampel H (2017). A precision medicine initiative for Alzheimer’s disease: the road ahead to biomarker-guided integrative disease modeling. Climacteric.

[CR55] Hanahan D, Weinberg RA (2000). The hallmarks of cancer. Cell.

[CR56] Hanahan D, Weinberg RA (2011). Hallmarks of cancer: the next generation. Cell.

[CR57] Hay M, Thomas DW, Craighead JL, Economides C, Rosenthal J (2014). Clinical development success rates for investigational drugs. Nat Biotechnol.

[CR58] Heilker R, Lessel U, Bischoff D (2018) The power of combining phenotypic and target-focused drug discovery. Drug Discov Today. 10.1016/j.drudis.2018.10.009 10.1016/j.drudis.2018.10.009 30359770

[CR59] Hoadley KA (2018). Cell-of-origin patterns dominate the molecular classification of 10,000 tumors from 33 types of cancer. Cell.

[CR60] Hodgkin J (1998). Seven types of pleiotropy. Int J Dev Biol.

[CR61] Hogeweg P (2011). The roots of bioinformatics in theoretical biology. PLoS Comput Biol.

[CR62] Houle D, Govindaraju DR, Omholt S (2010). Phenomics: the next challenge. Nat Rev Genet.

[CR63] Hunter FMI, LA F, Bento AP, Bosc N, Gaulton A, Hersey A, Leach AR (2018). A large-scale dataset of in vivo pharmacology assay results. Sci Data.

[CR64] Hyman DM, Taylor BS, Baselga J (2017). Implementing genome-driven oncology. Cell.

[CR65] Ioannidis JP (2005). Why most published research findings are false. PLoS Med.

[CR66] Ittisoponpisan S, Alhuzimi E, Sternberg MJ, David A (2017). Landscape of pleiotropic proteins causing human disease: structural and system biology insights. Hum Mutat.

[CR67] Jacoby E, Brown JB (2018). The future of computational chemogenomics. Methods Mol Biol.

[CR68] Jang H, Banerjee A, Chavan TS, Lu S, Zhang J, Gaponenko V, Nussinov R (2016). The higher level of complexity of K-Ras4B activation at the membrane. FASEB J.

[CR69] Jang H, Muratcioglu S, Gursoy A, Keskin O, Nussinov R (2016). Membrane-associated Ras dimers are isoform-specific: K-Ras dimers differ from H-Ras dimers. Biochem J.

[CR70] Jenkins KA (2018). The consequences of cavity creation on the folding landscape of a repeat protein depend upon context. Proc Natl Acad Sci U S A.

[CR71] Joslin J (2018). A fully automated high-throughput flow cytometry screening system enabling phenotypic drug discovery. SLAS Discov.

[CR72] Knievel J (2014). Multiple mechanisms mediate resistance to sorafenib in urothelial cancer. Int J Mol Sci.

[CR73] Knijnenburg TA (2018). Genomic and molecular landscape of DNA damage repair deficiency across The Cancer Genome Atlas. Cell Rep.

[CR74] Kumar S, Ma B, Tsai CJ, Sinha N, Nussinov R (2000). Folding and binding cascades: dynamic landscapes and population shifts. Protein Sci.

[CR75] Lage OM, Ramos MC, Calisto R, Almeida E, Vasconcelos V, Vicente F (2018). Current screening methodologies in drug discovery for selected human diseases. Mar Drugs.

[CR76] Lagunin AA (2018). CLC-Pred: a freely available web-service for in silico prediction of human cell line cytotoxicity for drug-like compounds. PLoS One.

[CR77] Lane T (2018). Comparing and validating machine learning models for Mycobacterium tuberculosis drug discovery. Mol Pharm.

[CR78] Lisman JE, Zhabotinsky AM (2001). A model of synaptic memory: a CaMKII/PP1 switch that potentiates transmission by organizing an AMPA receptor anchoring assembly. Neuron.

[CR79] Liu J, Nussinov R (2008). Allosteric effects in the marginally stable von Hippel-Lindau tumor suppressor protein and allostery-based rescue mutant design. Proc Natl Acad Sci U S A.

[CR80] Lu S, Banerjee A, Jang H, Zhang J, Gaponenko V, Nussinov R (2015). GTP binding and oncogenic mutations may attenuate hypervariable region (HVR)-catalytic domain interactions in small GTPase K-Ras4B, exposing the effector binding site. J Biol Chem.

[CR81] Lu S, Jang H, Muratcioglu S, Gursoy A, Keskin O, Nussinov R, Zhang J (2016). Ras conformational ensembles, allostery, and signaling. Chem Rev.

[CR82] Lu S, Jang H, Nussinov R, Zhang J (2016). The structural basis of oncogenic mutations G12, G13 and Q61 in small GTPase K-Ras4B. Sci Rep.

[CR83] Ma B, Nussinov R (2009). Amplification of signaling via cellular allosteric relay and protein disorder. Proc Natl Acad Sci U S A.

[CR84] Marcus K, Mattos C (2015). Direct attack on RAS: intramolecular communication and mutation-specific effects. Clin Cancer Res.

[CR85] Markossian S, Ang KK, Wilson CG, Arkin MR (2018). Small-molecule screening for genetic diseases. Annu Rev Genomics Hum Genet.

[CR86] Martin SD, Coukos G, Holt RA, Nelson BH (2015). Targeting the undruggable: immunotherapy meets personalized oncology in the genomic era. Ann Oncol.

[CR87] Mathew JP (2007). From bytes to bedside: data integration and computational biology for translational cancer research. PLoS Comput Biol.

[CR88] Merlo LM, Pepper JW, Reid BJ, Maley CC (2006). Cancer as an evolutionary and ecological process. Nat Rev Cancer.

[CR89] Mickert MJ, Gorris HH (2018). Transition-state ensembles navigate the pathways of enzyme catalysis. J Phys Chem B.

[CR90] Mills RM (2016) Why do drugs in development “fail” in clinical trials? https://www.pharmpro.com/article/2016/12/why-do-drugs-development-fail-clinical-trials/. Accessed Dec 12 2016

[CR91] Morra G, Verkhivker G, Colombo G (2009). Modeling signal propagation mechanisms and ligand-based conformational dynamics of the Hsp90 molecular chaperone full-length dimer. PLoS Comput Biol.

[CR92] Munos B (2009). Lessons from 60 years of pharmaceutical innovation. Nat Rev Drug Discov.

[CR93] Munro D, Ghersi D, Singh M (2018). Two critical positions in zinc finger domains are heavily mutated in three human cancer types. PLoS Comput Biol.

[CR94] Naganathan AN (2018). Modulation of allosteric coupling by mutations: from protein dynamics and packing to altered native ensembles and function. Curr Opin Struct Biol.

[CR95] Nakagawa H, Fujita M (2018). Whole genome sequencing analysis for cancer genomics and precision medicine. Cancer Sci.

[CR96] Nakamura A (2013). Antitumor activity of the selective pan-RAF inhibitor TAK-632 in BRAF inhibitor-resistant melanoma. Cancer Res.

[CR97] Nguemaha V, Qin S, Zhou HX (2018) Atomistic modeling of intrinsically disordered proteins under polyethylene glycol crowding: quantitative comparison with experimental data and implication of protein-Crowder attraction. J Phys Chem B. 10.1021/acs.jpcb.8b07066 10.1021/acs.jpcb.8b07066 PMC6447477 30230839

[CR98] Nikolaev SI (2018). Somatic activating KRAS mutations in arteriovenous malformations of the brain. N Engl J Med.

[CR99] Ninfa AJ, Mayo AE (2004). Hysteresis vs. graded responses: the connections make all the difference. Sci STKE.

[CR100] Nussinov R (2016). Introduction to protein ensembles and allostery. Chem Rev.

[CR101] Nussinov R, Tsai CJ (2013). Allostery in disease and in drug discovery. Cell.

[CR102] Nussinov R, Tsai CJ (2015). ‘Latent drivers’ expand the cancer mutational landscape. Curr Opin Struct Biol.

[CR103] Nussinov R, Wolynes PG (2014). A second molecular biology revolution? The energy landscapes of biomolecular function. Phys Chem Chem Phys.

[CR104] Nussinov R, Tsai CJ, Csermely P (2011). Allo-network drugs: harnessing allostery in cellular networks. Trends Pharmacol Sci.

[CR105] Nussinov R, Tsai CJ, Xin F, Radivojac P (2012). Allosteric post-translational modification codes. Trends Biochem Sci.

[CR106] Nussinov R, Jang H, Tsai CJ (2014). The structural basis for cancer treatment decisions. Oncotarget.

[CR107] Nussinov R, Tsai CJ, Chakrabarti M, Jang H (2016). A new view of Ras isoforms in cancers. Cancer Res.

[CR108] Nussinov R, Jang H, Tsai CJ, Liao TJ, Li S, Fushman D, Zhang J (2017). Intrinsic protein disorder in oncogenic KRAS signaling. Cell Mol Life Sci.

[CR109] Nussinov R, Tsai CJ, Jang H (2018). Oncogenic Ras isoforms signaling specificity at the membrane. Cancer Res.

[CR110] Nussinov R, Zhang M, Tsai CJ, Liao TJ, Fushman D, Jang H (2018). Autoinhibition in Ras effectors Raf, PI3Kalpha, and RASSF5: a comprehensive review underscoring the challenges in pharmacological intervention. Biophys Rev.

[CR111] Oberg V, Differding J, Fisher M, Hines L, Wilke RA (2016). Navigating pleiotropy in precision medicine: pharmacogenes from trauma to behavioral health. Pharmacogenomics.

[CR112] Orellana A (2018). Application of a phenotypic drug discovery strategy to identify biological and chemical starting points for inhibition of TSLP production in lung epithelial cells. PLoS One.

[CR113] Ortiz D (2017). Discovery of novel, orally bioavailable, antileishmanial compounds using phenotypic screening. PLoS Negl Trop Dis.

[CR114] Pantsar T, Rissanen S, Dauch D, Laitinen T, Vattulainen I, Poso A (2018). Assessment of mutation probabilities of KRAS G12 missense mutants and their long-timescale dynamics by atomistic molecular simulations and Markov state modeling. PLoS Comput Biol.

[CR115] Park MJ (2018). Oncogenic exon 2 mutations in mediator subunit MED12 disrupt allosteric activation of cyclin C-CDK8/19. J Biol Chem.

[CR116] Payne PR (2012). Chapter 1: biomedical knowledge integration. PLoS Comput Biol.

[CR117] Pendergrass SA, Ritchie MD (2015). Phenome-wide association studies: leveraging comprehensive phenotypic and genotypic data for discovery. Curr Genet Med Rep.

[CR118] Peng X (2018). Molecular characterization and clinical relevance of metabolic expression subtypes in human cancers. Cell Rep.

[CR119] Perrin S (2014). Preclinical research: make mouse studies work. Nature.

[CR120] Poulikakos PI, Zhang C, Bollag G, Shokat KM, Rosen N (2010). RAF inhibitors transactivate RAF dimers and ERK signalling in cells with wild-type BRAF. Nature.

[CR121] Poulos RC, Wong JWH (2018) Finding cancer driver mutations in the era of big data research. Biophys Rev. 10.1007/s12551-018-0415-6 10.1007/s12551-018-0415-6 PMC6381364 29611034

[CR122] Prasad V (2016). Perspective: the precision-oncology illusion. Nature.

[CR123] Prehoda KE, Lim WA (2002). How signaling proteins integrate multiple inputs: a comparison of N-WASP and Cdk2. Curr Opin Cell Biol.

[CR124] Prinz F, Schlange T, Asadullah K (2011). Believe it or not: how much can we rely on published data on potential drug targets?. Nat Rev Drug Discov.

[CR125] Qiao W, Akhter N, Fang X, Maximova T, Plaku E, Shehu A (2018). From mutations to mechanisms and dysfunction via computation and mining of protein energy landscapes. BMC Genomics.

[CR126] Raphael BJ (2012). Chapter 6: structural variation and medical genomics. PLoS Comput Biol.

[CR127] Raphael BJ, Dobson JR, Oesper L, Vandin F (2014). Identifying driver mutations in sequenced cancer genomes: computational approaches to enable precision medicine. Genome Med.

[CR128] Riddy DM, Goy E, Delerive P, Summers RJ, Sexton PM, Langmead CJ (2018). Comparative genotypic and phenotypic analysis of human peripheral blood monocytes and surrogate monocyte-like cell lines commonly used in metabolic disease research. PLoS One.

[CR129] Risques RA, Kennedy SR (2018). Aging and the rise of somatic cancer-associated mutations in normal tissues. PLoS Genet.

[CR130] Roskoski R (2014). The ErbB/HER family of protein-tyrosine kinases and cancer. Pharmacol Res.

[CR131] Rowland MA, Greenbaum JM, Deeds EJ (2017). Crosstalk and the evolvability of intracellular communication. Nat Commun.

[CR132] Ruan Z, Kannan N (2018). Altered conformational landscape and dimerization dependency underpins the activation of EGFR by alphaC-beta4 loop insertion mutations. Proc Natl Acad Sci U S A.

[CR133] Rukhlenko OS (2018). Dissecting RAF inhibitor resistance by structure-based modeling reveals ways to overcome oncogenic RAS signaling. Cell Syst.

[CR134] Rychkov GN (2017). Partially assembled nucleosome structures at atomic detail. Biophys J.

[CR135] Sanchez-Vega F (2018). Oncogenic signaling pathways in The Cancer Genome Atlas. Cell.

[CR136] Scannell JW, Bosley J (2016). When quality beats quantity: decision theory, drug discovery, and the reproducibility crisis. PLoS One.

[CR137] Scannell JW, Blanckley A, Boldon H, Warrington B (2012). Diagnosing the decline in pharmaceutical R&D efficiency. Nat Rev Drug Discov.

[CR138] Schaub FX (2018). Pan-cancer alterations of the MYC oncogene and its proximal network across The Cancer Genome Atlas. Cell Syst.

[CR139] Scheeder C, Heigwer F, Boutros M (2018). Machine learning and image-based profiling in drug discovery. Curr Opin Syst Biol.

[CR140] Schurdak ME, Pei F, Lezon TR, Carlisle D, Friedlander R, Taylor DL, Stern AM (2018). A quantitative systems pharmacology approach to infer pathways involved in complex disease phenotypes. Methods Mol Biol.

[CR141] Senft D, Leiserson MDM, Ruppin E, Ronai ZA (2017). Precision oncology: the road ahead. Trends Mol Med.

[CR142] Shen Q (2017). Proteome-scale investigation of protein allosteric regulation perturbed by somatic mutations in 7,000 cancer genomes. Am J Hum Genet.

[CR143] Sidders B, Karlsson A, Kitching L, Torella R, Karila P, Phelan A (2018). Network-based drug discovery: coupling network pharmacology with phenotypic screening for neuronal excitability. J Mol Biol.

[CR144] Solovieff N, Cotsapas C, Lee PH, Purcell SM, Smoller JW (2013). Pleiotropy in complex traits: challenges and strategies. Nat Rev Genet.

[CR145] Stearns FW (2010). One hundred years of pleiotropy: a retrospective. Genetics.

[CR146] Steklov M et al (2018) Mutations in LZTR1 drive human disease by dysregulating RAS ubiquitination. Science. 10.1126/science.aap7607 10.1126/science.aap7607 PMC8058620 30442762

[CR147] Stiewe T, Haran TE (2018). How mutations shape p53 interactions with the genome to promote tumorigenesis and drug resistance. Drug Resist Updat.

[CR148] Stout MC, Campbell PM (2018). RASpecting the oncogene: new pathways to therapeutic advances. Biochem Pharmacol.

[CR149] Stratton MR, Campbell PJ, Futreal PA (2009). The cancer genome. Nature.

[CR150] Szilagyi A, Nussinov R, Csermely P (2013). Allo-network drugs: extension of the allosteric drug concept to protein- protein interaction and signaling networks. Curr Top Med Chem.

[CR151] Tannock IF, Hickman JA (2016). Limits to personalized cancer medicine. N Engl J Med.

[CR152] Tehver R, Chen J, Thirumalai D (2009). Allostery wiring diagrams in the transitions that drive the GroEL reaction cycle. J Mol Biol.

[CR153] Thiagalingam S (1996). Evaluation of candidate tumour suppressor genes on chromosome 18 in colorectal cancers. Nat Genet.

[CR154] Tomczak K, Czerwinska P, Wiznerowicz M (2015). The Cancer Genome Atlas (TCGA): an immeasurable source of knowledge. Contemp Oncol (Pozn).

[CR155] Tsai CJ, Nussinov R (2014). The free energy landscape in translational science: how can somatic mutations result in constitutive oncogenic activation?. Phys Chem Chem Phys.

[CR156] Tsai CJ, Nussinov R (2017). Allostery modulates the beat rate of a cardiac pacemaker. J Biol Chem.

[CR157] Tsai CJ, Nussinov R (2018). Allosteric activation of RAF in the MAPK signaling pathway. Curr Opin Struct Biol.

[CR158] Tsai CJ, Del Sol A, Nussinov R (2009). Protein allostery, signal transmission and dynamics: a classification scheme of allosteric mechanisms. Mol BioSyst.

[CR159] Tsang H, Addepalli K, Davis SR (2017). Resources for interpreting variants in precision genomic oncology applications. Front Oncol.

[CR160] Tuncbag N, Kar G, Gursoy A, Keskin O, Nussinov R (2009). Towards inferring time dimensionality in protein-protein interaction networks by integrating structures: the p53 example. Mol BioSyst.

[CR161] Tuncbag N, Keskin O, Nussinov R, Gursoy A (2017). Prediction of protein interactions by structural matching: prediction of PPI networks and the effects of mutations on PPIs that combines sequence and structural information. Methods Mol Biol.

[CR162] Tyler AL, Crawford DC, Pendergrass SA (2016). The detection and characterization of pleiotropy: discovery, progress, and promise. Brief Bioinform.

[CR163] Vaidya A, Roy A, Chaguturu R (2018). How to rekindle drug discovery process through integrative therapeutic targeting?. Expert Opin Drug Discovery.

[CR164] Vargas AJ, Harris CC (2016). Biomarker development in the precision medicine era: lung cancer as a case study. Nat Rev Cancer.

[CR165] Verkhivker GM (2019). Biophysical simulations and structure-based modeling of residue interaction networks in the tumor suppressor proteins reveal functional role of cancer mutation hotspots in molecular communication. Biochim Biophys Acta Gen Subj.

[CR166] Verma S, Goyal S, Kumari A, Singh A, Jamal S, Grover A (2018). Structural investigations on mechanism of lapatinib resistance caused by HER-2 mutants. PLoS One.

[CR167] Voest EE, Bernards R (2016). DNA-guided precision medicine for cancer: a case of irrational exuberance?. Cancer Discov.

[CR168] Vogelstein B, Papadopoulos N, Velculescu VE, Zhou S, Diaz LA, Kinzler KW (2013). Cancer genome landscapes. Science.

[CR169] Wagner GP, Zhang J (2011). The pleiotropic structure of the genotype-phenotype map: the evolvability of complex organisms. Nat Rev Genet.

[CR170] Wang Z, Liao BY, Zhang J (2010). Genomic patterns of pleiotropy and the evolution of complexity. Proc Natl Acad Sci U S A.

[CR171] Waters AM, Der CJ (2018). KRAS: the critical driver and therapeutic target for pancreatic cancer. Cold Spring Harb Perspect Med.

[CR172] Way GP (2018). Machine learning detects pan-cancer Ras pathway activation in The Cancer Genome Atlas. Cell Rep.

[CR173] Wei G, Xi W, Nussinov R, Ma B (2016). Protein ensembles: how does nature harness thermodynamic fluctuations for life? The diverse functional roles of conformational ensembles in the cell. Chem Rev.

[CR174] Welch JS (2012). The origin and evolution of mutations in acute myeloid leukemia. Cell.

[CR175] Welcome to the pan-cancer atlas (2016) https://www.cell.com/pb-assets/consortium/pancanceratlas/pancani3/index.html. Accessed May 22 2018

[CR176] Winter C (2012). Google goes cancer: improving outcome prediction for cancer patients by network-based ranking of marker genes. PLoS Comput Biol.

[CR177] Wymann MP, Schneiter R (2008). Lipid signalling in disease. Nat Rev Mol Cell Biol.

[CR178] Xu Q (2017). Benchmarking predictions of allostery in liver pyruvate kinase in CAGI4. Hum Mutat.

[CR179] Yakhini Z, Jurisica I (2011). Cancer computational biology. BMC Bioinformatics.

[CR180] Youn A, Simon R (2011). Identifying cancer driver genes in tumor genome sequencing studies. Bioinformatics.

[CR181] Yu L, Li K, Zhang X (2017). Next-generation metabolomics in lung cancer diagnosis, treatment and precision medicine: mini review. Oncotarget.

[CR182] Zhan C, Qi R, Wei G, Guven-Maiorov E, Nussinov R, Ma B (2016). Conformational dynamics of cancer-associated MyD88-TIR domain mutant L252P (L265P) allosterically tilts the landscape toward homo-dimerization. Protein Eng Des Sel.

